# Analytical Ultracentrifugation in Different High‐Density Media Allows to Assess Heterogeneity of mRNA‐Lipid Nanoparticles

**DOI:** 10.1002/smtd.202502420

**Published:** 2026-06-26

**Authors:** Dora Mehn, Mariana Hugo Silva, Rein Verbeke, Allegra Peletta, Miffy Hok Yan Cheng, Stefaan C. De Smedt, Silvia Lucia Appleton, Ambra Sarracino, Jessica Ponti, Luigi Calzolai, Ine Lentacker

**Affiliations:** ^1^ European Commission Joint Research Centre (JRC) Ispra Italy; ^2^ Ghent Research group on Nanomedicines, Labo of General Biochemistry and Physical Pharmacy Ghent University Ghent Belgium; ^3^ Faculty of Pharmaceutical Sciences Vancouver British Columbia Canada

**Keywords:** analytical ultracentrifugation, AUC, lipid nanoparticle, LNP, sedimentation velocity

## Abstract

This study demonstrates the capability of analytical ultracentrifugation for assessing the morphological homogeneity of mRNA‐lipid nanoparticles. Building on a standard method for density evaluation of nanoparticles, it demonstrates the importance of selecting the appropriate liquid medium for the experiments. Our research reveals the interaction between deuterated buffers and aqueous compartments of lipid‐based particles. By investigating the sedimentation behavior of compact solid particles, liposomes, and lipid nanoparticles, we demonstrate that AUC can detect water‐loaded cavities in mRNA‐loaded lipid nanoparticles. Therefore, we suggest that performing sedimentation velocity experiments both in sucrose‐containing buffer and deuterated solvents results in precious information not only on density but also on batch homogeneity and particle morphology.

## Graphical abstract

We investigate the application of analytical ultracentrifugation for evaluating the homogeneity of mRNA‐lipid nanoparticles, exploring the effects of different sedimentation media on particle behavior, and demonstrating the technique's potential for characterizing nanoparticle density and detecting liquid‐loaded compartments.

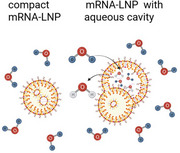

## 1 Introduction

Lipid‐based nanoparticles, including liposomes and lipid nanoparticle (LNP) formulations, account for approximately 50% of the global nanomedicine market share. LNPs, particularly for ribonucleic acid (RNA delivery, have experienced rapid growth in recent years due to their use in corona virus disease 2019 vaccines and other novel therapies [1, 2, 3]. Size characterization of lipid‐based nanoparticles is typically done using methods that measure the diffusion coefficient by dynamic light scattering or nanoparticle tracking analysis [4, 5, 6, 7, 8]. More advanced characterization to gain morphological and structural insights is possible with transmission electron microscopy (TEM)—using cryogenic‐TEM, as traditional TEM preparative methods could induce staining artifacts and compromise particles’ structural integrity in a vacuum environment [9, 10, 11]. This complicates the application of TEM imaging in their characterization. Other, higher throughput, more economical, and truly orthogonal characterization methods for nanomedicines in aqueous buffer are centrifugal sedimentation methods [12]. Because sedimentation speed depends not only on particle size, but also on density (and shape), these methods can also provide additional information on density. and might, therefore, be extremely useful during drug product development. For example, it was shown that the density of LNPs correlates with their RNA content [13, 14, 15, 16]. Line‐start centrifugal methods, where the particle suspension is injected on top of a density gradient, are less commonly used for lipid‐based nanoparticles than for inorganic ones, primarily due to the low particle density. The density of 0.85–1.05 g/mL limits or completely impedes the measurement of sedimentation in a sucrose gradient, typically applied in line‐start centrifugal liquid sedimentation instruments (i.e., disc centrifuges). In this type of method, the particles should sediment from the top of the gradient in the direction of the detector located close to the perimeter of the disc in function of density [17]. Density determination of liposomes by centrifugal field flow fractionation has been reported, but not all commercial instruments can achieve the necessary rotational speed for reasonable separation in these experiments [18]. In contrast, this is the case for analytical ultracentrifugation (AUC) making this method well‐suited for performing sedimentation velocity measurements, enabling easy comparison of batches based on their sedimentation coefficient distribution in native buffer. Moreover, this allows to estimate density of nanoparticles, for example, by using dynamic light scattering (DLS) diameter as hydrodynamic size or a density matching approach [19, 20, 21, 22]. AUC is a well‐established and validated technique for nanoparticle characterization, and an ISO (International Organisation for Standardisation) standard exists for density calculations, outlining the theoretical considerations and practical solutions [23]. The fundamental principle underlying this method is that when both particle size and density are unknown, sedimentation speed is measured in two liquid media of different densities. This allows calculation of both values using the Stokes equation when liquid phase densities and viscosities are known (Equations (1) and (2)). According to the ISO document, D_2_O is recommended as a second solvent instead of water, which is particularly effective for inorganic particles. Moreover, D_2_O has also been found to be a suitable component in higher‐density PBS (phosphate buffered saline)/D_2_O mixtures for LNPs [13]. However, D_2_O can interact with systems containing water molecules (e.g., hydration layers) or with easily exchangeable hydrogen atoms in their structure (such as poly‐alcohols), thus compromising the direct applicability of D_2_O‐based solvents for various nanomedicine particles. Our work shows that this is the case for phase‐separated LNP structures containing aqueous cavities and/or blebs. While this may appear to be a limitation when selecting an appropriate high‐density buffer for sedimentation velocity experiments, our data show that this provides an opportunity to detect phase separation in mRNA‐loaded LNPs when aqueous cavities are formed.

## 2 Results and Discussion

### 2.1 Sedimentation Behavior of Compact, Solid Particles

**FIGURE 1:**
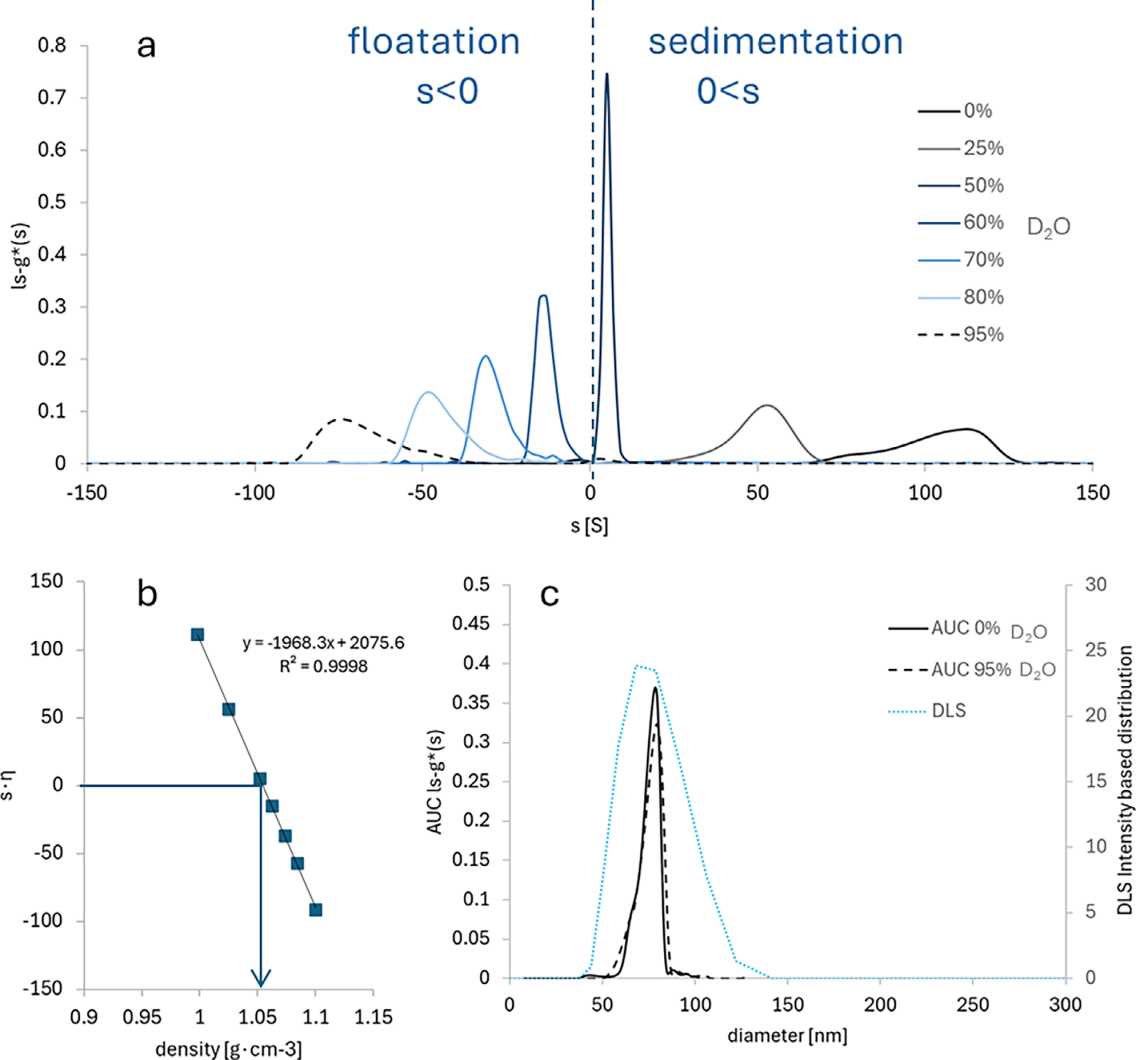
(a) Sedimentation coefficient distribution of PS nanoparticles in various H_2_O/D_2_O mixtures with different densities (*n* = 1, fit confidence level 0.95); (b) Linear dependence of viscosity‐corrected sedimentation coefficient as a function of liquid density, illustrating that the sedimentation coefficient becomes zero when particle density matches the liquid density; (c) Comparison of size distribution results measured at the two density extremes (in 95% D_2_O and in H_2_O corresponding to densities of 1.100 and 0.998 g·cm^−3^, respectively) by AUC and measured in water by DLS (*n* = 3).

Polystyrene (PS) beads were used to illustrate the sedimentation behavior of compact, solid particles in sedimentation velocity experiments. These particles are reasonably monodisperse and are commercially available in a size range relevant to nanomedicine products. In centrifugation velocity experiments, these particles sedimented in water and floated in 95% D_2_O. Figure 1a illustrates their behavior in various D_2_O:H_2_O mixtures. With the increasing density of the liquid medium, the mode of the sedimentation coefficient (*s*) distribution is shifted to lower values (Figure 1a and Table S1). The detected sedimentation coefficient distribution peak is narrower when the mode is closer to zero (particle density is close to that of the medium) and wider when the difference between liquid and particle density is larger. The viscosity corrected sedimentation velocity shows an excellent linear correlation with liquid density across the entire investigated range (Figure 1b), suggesting that any two measurement points could be applied to determine the density of these particles according to Equation (3) (Section 4. Experimental methods). At the liquid density that matches the particle density, the sedimentation velocity becomes zero (Figure 1b). Size distributions calculated from the various sedimentation coefficient distributions using a single density value (1.054 gcm^−3^) show a very good overlap, even at the two liquid density extremes (in water and in 95% D_2_O), as illustrated in Figure 1c. This also confirms that the inhomogeneity (peak shoulder) observed in the centrifugal sedimentation experiments is primarily due to the presence of various size particle populations and not—or in a much smaller extent – due to the variability in density. The size distribution is comparable to the distribution measured by DLS (Figure 1c), although AUC provides better resolution.

### 2.2 Sedimentation Behavior of Liposomes in Various Buffers

**FIGURE 2:**
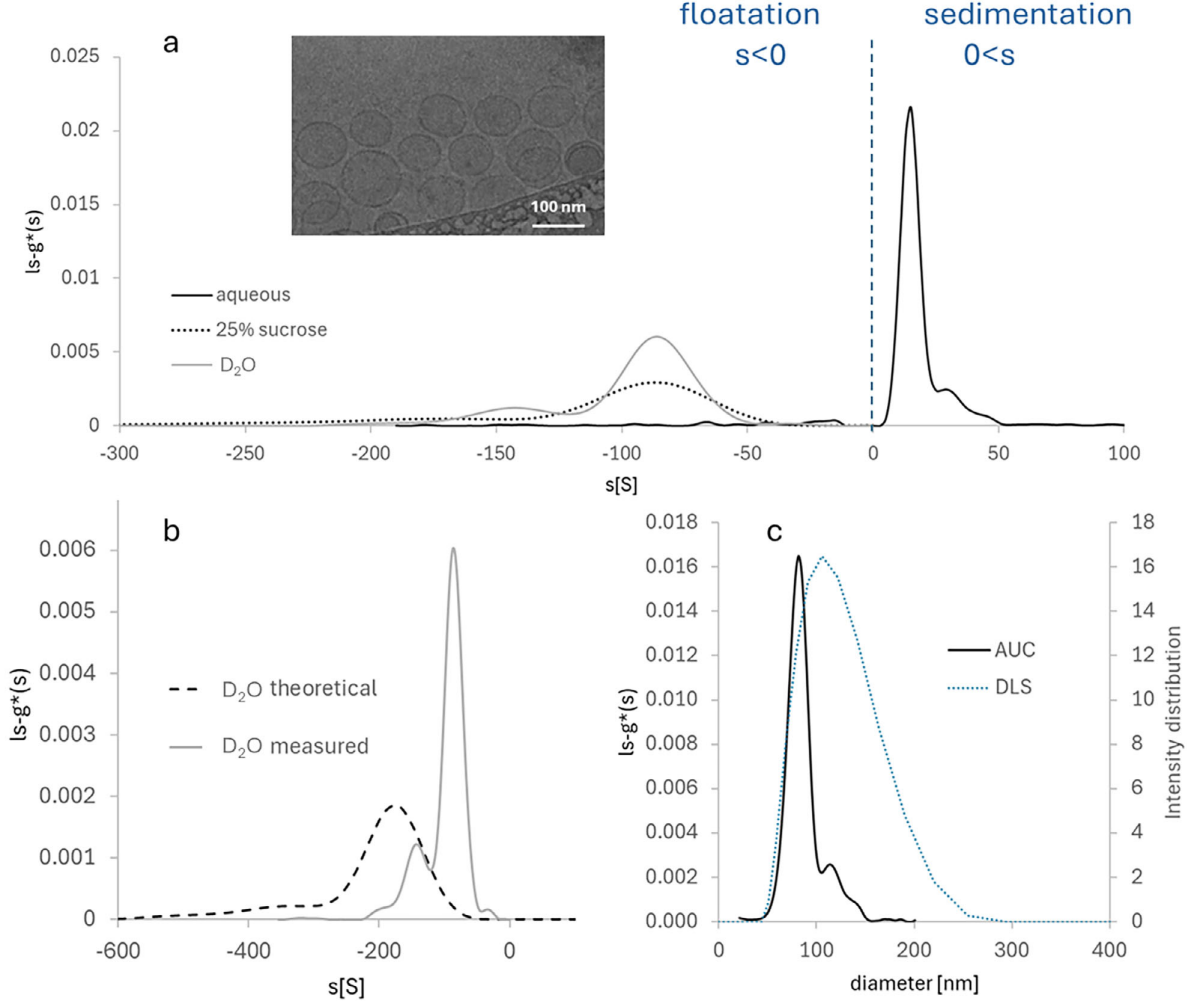
(a) Sedimentation coefficient distribution of liposomes in aqueous buffer (solid, black line), 25% sucrose solution (dotted line), and D_2_O‐based buffer (gray line); (b) Sedimentation coefficient distribution of liposomes measured in D_2_O‐based buffer (gray line) compared to the theoretically expected distribution calculated from the sedimentation profile in aqueous buffer (dashed line). (*n* = 3, fit confidence level 0.95) Note that the measurement in D_2_O is the same as in Figure 2a, only scales are different; (c) Hydrodynamic size distribution calculated from AUC in aqueous buffer and in sucrose‐containing medium (solid, black line) compared to the size distribution measured with DLS (dotted blue line, *n* = 3). The insert shows a Cryo‐TEM image of the sample.

The PEGylated liposomes used in this study were produced and stored in‐house in phosphate‐buffered saline solution (PBS) (Section 4. Experimental methods). They appear unilamellar in Cryo‐TEM images (Figure 2a, insert). Sedimentation experiments were conducted in PBS, PBS prepared in D_2_O, and 25% sucrose containing PBS, in order to maintain the same salt concentration. The liposomes sedimented in aqueous PBS solution and floated in 25% sucrose/PBS (Figure 2a). Their density, determined from the ± sucrose/PBS experiments (according to Equation (3) and considering the main peak modes), is 1.005 g·cm^−3^. The main population has a slightly smaller size (with a mode at 82 nm) than the intensity‐based hydrodynamic diameter distribution measured by DLS, where the PEGylated liposomes showed an intensity‐based distribution mode of approximately 106 nm (Figure 2c). The two distributions are in reasonable agreement (Figure 2c), considering the inherent limitation of DLS, a bulk method in which larger particles can obscure smaller ones due to their higher scattering intensity.

On the other hand, the calculated density is higher (1.011 g·cm^−3^) when using the floatation speed detected in the deuterated solvent. Applying this density to transform the sedimentation data into a particle size distribution would yield a much smaller diameter (approximately 60 nm) than the one measured with DLS. This non‐ideal behavior in D_2_O can also be visualized by comparing the calculated, theoretically predicted *s*‐distribution (see 4. Experimental Methods/Calculations) with the measured one (Figure 2b). The mode of the measured distribution in the deuterated liquid appears at a lower absolute value than expected. The most probable explanation is the interaction of the liposomes with the liquid medium. If D_2_O‐H_2_O (or deuterium‐hydrogen ion) exchange occurs between the surrounding liquid and the internal aqueous compartment of the liposomes, the density of the liposomes increases during the experiment. This leads to a lower‐than‐expected floatation speed and a mismatch between the Stokes diameter calculated from centrifugal sedimentation and the hydrodynamic diameter measured by DLS.

The observed D_2_O‐H_2_O (or deuterium‐hydrogen ion) exchange across lipid bilayer membranes is not unexpected. Although the water content of lipid bilayers is very low—the hydrophobic core is reported to contain about one water molecule per lipid molecule, and surprisingly fast diffusion of water and H^+^ ion through lipid bilayers is a well‐documented phenomenon [24] known to depend on membrane composition [25]. While the presence of cholesterol and saturated fatty acid chains is generally thought to decrease water permeability, cholesterol has been shown to increase H^+^ flux [26, 27]. The diffusion coefficient of water molecules through the hydrophobic core of the bilayer is estimated to be only three orders of magnitude lower than that of free water molecules [28]. D_2_O and D^+^ ions are expected to behave similarly to H_2_O and H^+^ ions and pass through the membrane from the buffer medium to the internal cavity of the liposomes.

Moreover, the density of these liposomes, corresponding to a sedimentation coefficient of −85 S in the deuterated solvent, is approximately 1.074 g·cm^−3^. This represents a 6.8% increase in density compared to the apparent density calculated from the sedimentation coefficient in H_2_O, which equals the percentage increase in mass – assuming the liposome volume remains unchanged (Equation (5)). Indeed, when considering an aqueous core of 70 nm (with a lipid bilayer thickness of 5–6 nm) and replacing all H_2_O molecules in the core with D_2_O, the estimated particle mass increase is about 6.7%—in good agreement with the density increase (Equations (6), (7), (8), (9), (10), (11)). This suggests that the majority of H_2_O molecules of the core and most probably also those of the hydration layer are exchanged for D_2_O.

Similar behavior and mass increase of particles was observed in H_2_‐^18^O water as shown in the (Figure S2). This confirms the hypothesis that not only D^+^ and H^+^ ions, but also water molecules are able to diffuse through the lipid bilayer, allowing the complete exchange of the aqueous cavity to reach equilibrium with the external aqueous phase in a very short time.

Looking at the good agreement between the DLS and AUC‐based size distributions, the density determined from the ± sucrose/PBS experiments can be considered reliable. These results indicate that sucrose does not permeate the lipid bilayer of the PEGylated liposomes. Sucrose, as well as other small sugar molecules such as trehalose, are cryo‐protectants and well‐known to stabilize lipid bilayers [29, 30, 31]. Moreover, sucrose solutions have been shown to be suitable for density determination of drug‐loaded liposomes such as Doxil, using density match type experiments [22].

Additionally, comparison with Nanoparticle Tracking Analysis results (NTA) shows that AUC might be more powerful in detecting low scatterer and/or small diameter particle populations that fall under the detection limit of NTA (Figures S3 and S4).

### 2.3 Sedimentation Behavior of mRNA‐Loaded Lipid Nanoparticles (mRNA‐LNPs) in Various Buffers

**FIGURE 3:**
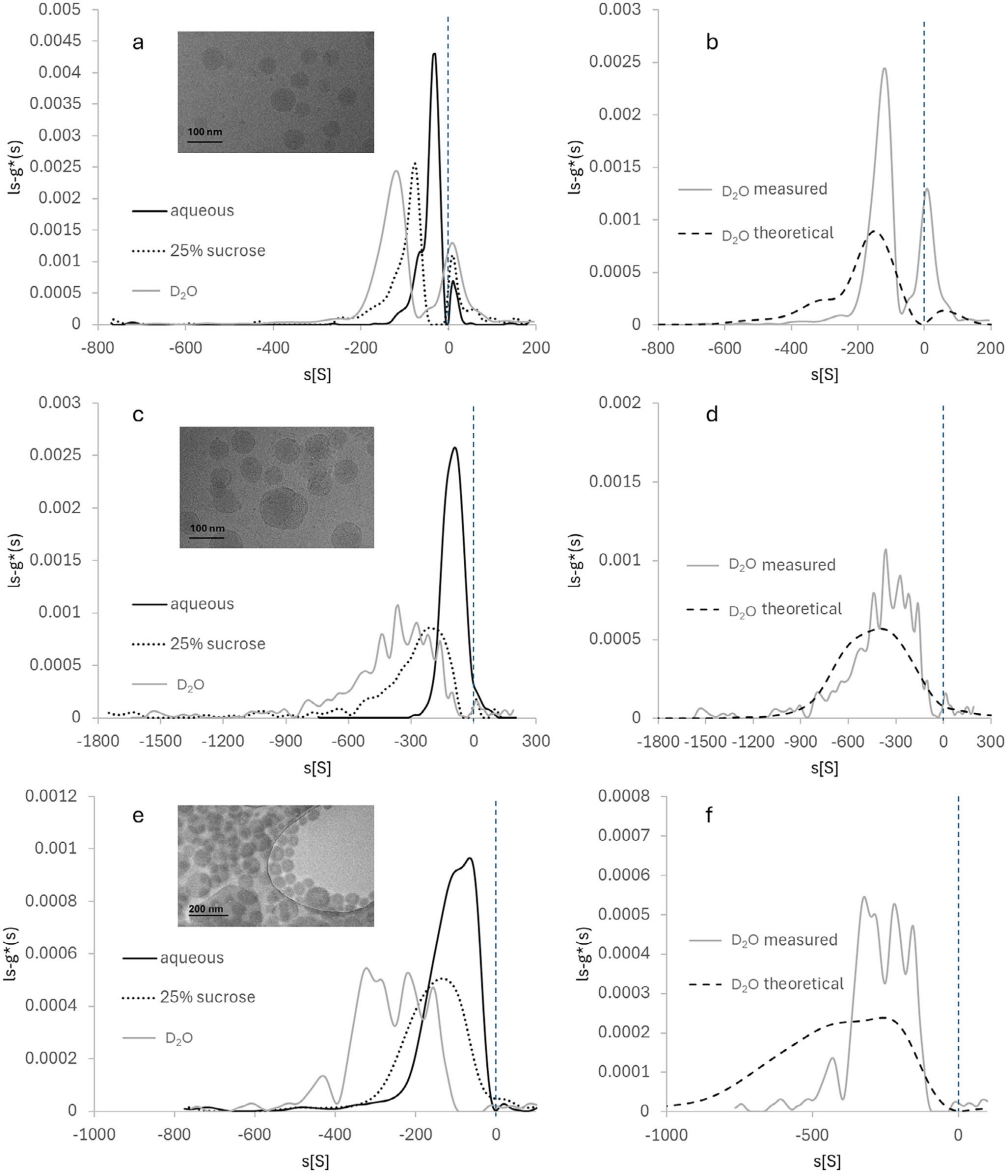
Sedimentation behavior of (a) intact and compact mRNA LNPs and (c) mRNA LNPs with blebs and e) aqueous cavity containing lower‐density mRNA LNPs in various liquids (solid, black line – PBS, gray line – D_2_O/PBS, dotted line – 25% sucrose in PBS) (*n* = 1–3, fit confidence level 0.95). Figures b, d, f illustrate the theoretically expected sedimentation coefficient distributions (dashed lines) compared to the measured ones (gray lines) in deuterated solvent, highlighting the non‐idealistic behavior in the case of particles with aqueous compartments. Measured distributions (in gray) appear at less negative s values in the case of the particles with water‐loaded cavities because of the density increase in D_2_O/PBS. Inserts show cryo‐TEM images of the particles. Dashed, vertical blue lines highlight the zero sedimentation coefficient value – above 0 S particles sediment, below 0 S particles float.

Figure 3 illustrates the sedimentation behavior and morphology (Cryo‐TEM image inserts) of the LNPs investigated in this study. The density of mRNA‐LNPs is very close to that of water but still dependent on their mRNA load [13]. They typically float in PBS and float even faster in media with higher densities, such as deuterated solvents [13, 15] or 25% sucrose/PBS (Figure 3).

Compact, nearly ideal mRNA LNPs – even if they contain some internal water [32]‐ behave very similarly to PS particles (Figures 1a and 3a, respectively). Their dense, hydrophobic core is less accessible to solvent molecules, and the PEGylated surface stabilizes the particles, allowing the use of both sucrose‐containing media, as well as D_2_O‐based solvents for AUC. Their density and size can be well estimated based on AUC experiments run at various D_2_O concentrations as previously suggested by Parrot et al. [13]. When comparing the theoretically predicted sedimentation coefficient distribution in D_2_O (see 4. Experimental Methods/Calculations) with the experimentally measured one, the modes of the two distributions show very similar s‐values (about −120 S in Figure 3d), although the theoretical distribution is slightly broader. The imperfect overlap between the peaks is most likely due to density heterogeneity within the LNP population, for example larger ones might have slightly higher densities than smaller ones because of a larger mRNA load [13]. Depending on their composition or production method [33], or upon destabilization mRNA LNPs often appear less compact and less spherical in cryo‐EM images [34]. Such particles frequently exhibit aqueous compartments in the form of internal cavities or are surrounded by uni‐ or multilamellar membranes attached to a hydrophobic, lipid‐rich core. Figure 3b,c illustrates the behavior of these less compact LNPs. They float in both aqueous and 25% sucrose containing buffers, but display a broader sedimentation coefficient distribution shifted to more negative sedimentation coefficient values in the presence of sucrose because of the higher density of the medium.

**FIGURE 4:**
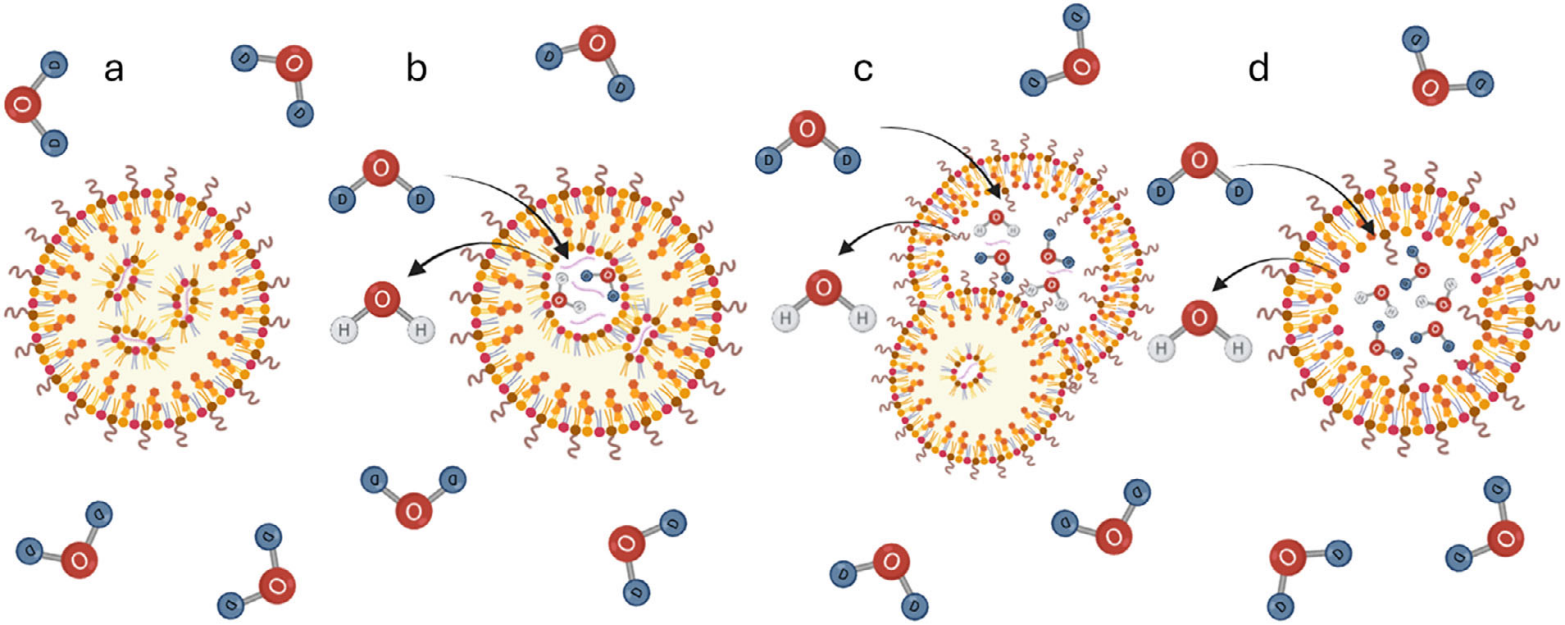
Schematic drawing of D_2_O and H_2_O exchange between solvent and water‐loaded cavities in lipid‐based particles when (a) no detectable solvent exchange occurs in compact mRNA‐LNPs, while D_2_O enters water‐loaded cavities in (b,c) phase‐separated mRNA‐LNPs and (d) liposomes. Created in BioRender. Mehn, D. (2026). Https://BioRender.com/cmynnhs.

In deuterated solvent, the particle populations that initially appeared monomodal resolve into multiple subpopulations (Figure 3c,e), floating at different velocities, some of which exhibit a lower velocity than expected from the theoretical calculation (gray compared to dashed black lines in Figure 3d,f). Based on our observations with liposomes, we propose that this is a result of a similar interaction between the sedimentation medium and the internal aqueous compartments of the LNPs. We hypothesize that exchange of solvent molecules, or their deuterium and hydrogen ions, alters the particle density (as illustrated in Figure 4) . The extent of this density change depends on the relative volumes of the hydrophobic, lipid‐rich core and the water‐filled compartments and is likely further influenced by particle lamellarity and geometry. The solvent‐induced increase in particle density leads to slower flotation, reflected by a shift of the measured sedimentation coefficient distribution*s* toward less negative values (gray lines in Figure 3d,f) compared with the theoretically expected distributions (dashed lines in Figure 3d,f) .

The density determined from the ± sucrose/PBS measurements enables conversion of the sedimentation coefficient distribution into a size distribution. This derived size distribution is in good agreement with the intensity‐based distribution measured with DLS even in the case of less compact particles (Figure S1).

## 3 Conclusions

Characterizing lipid‐based nanomedicines using AUC requires special considerations compared to analyzing compact polymeric or metal‐based nanoparticles. Due to their fragility, sedimentation velocities must be moderate to avoid destructive shear forces. In this study, a speed of 10 000 rpm was applied, which was compatible with both liposomes and lipid nanoparticles – as shown by the good agreement between DLS and AUC size results.

As sedimentation velocity depends on both size and density, simultaneous measurements through orthogonal techniques such as DLS, or multiple AUC runs in various liquids are necessary to decouple these parameters. For multiple velocity AUC experiments, the native buffer of the particles is often the preferred sedimentation medium, as AUC experiments can be readily performed in these buffers with minimal sample preparation, typically requiring only simple dilution. Our results show that using a sucrose solution as a second sedimentation medium is preferable to a D_2_O‐based buffers, as sucrose does not permeate the lipid bilayer and therefore does not enter liposomes or LNPs, whereas deuterated solvents may interact with water‐filled compartments within these nanoparticles. On the other hand, such solvent‐particle interactions can be exploited experimentally. Specifically, differences observed between measurements in sucrose solution and deuterated solvents may reveal the presence of water‐loaded cavities and thus provide valuable insights into particle non‐homogeneity within the LNP population.

Sedimentation velocity is also influenced by particle shape, however, many nanomedicine products, including liposomes, emulsions, and lipid nanoparticles, can be reasonably approximated as spherical. Deviations from a compact, spherical particle morphology may be either desirable or undesirable depending on the final application [35]. While cryo‐TEM remains the gold standard for visualizing morphological differences, our results indicate that AUC is a promising method to detect distinct morphologies and can provide valuable information on particle homogeneity, and could serve as a useful tool in particle stability and storage condition testing studies. The method also has clear limitations: although D_2_O ‐water exchange reveals sample heterogeneity, the simultaneous presence of size, shape, and compositional variations in mRNA LNPs prevents a reliable quantitative estimation of the internal aqueous‐volume fraction using the simple liposome model. Compared to cryo‐EM, AUC offers higher throughput and allows for the study of nanomedicines in their native buffer and in a liquid phase, without the need for freezing. Compared to NTA, AUC offers a tool that can also detect smaller, low‐scattering particle populations. Moreover, the use of higher‐density solvents, such as the sucrose solution presented here, can increase floatation velocities and reduce experiment duration, potentially enabling the use of lower‐speed and hence lower‐cost centrifugal methods.

## 4 Experimental Methods

### 4.1 Materials

All chemicals were purchased from Merck (Darmstadt, Germany) if not stated otherwise. Polystyrene standard beads were from Malvern Panalytical (Malvern, UK).

### 4.2 Synthesis of Lipid‐Based Particles

PEGylated liposomes were prepared using a microfluidic synthesizer (NanoAssemblr Ignite, Cytiva). Mixing of an organic phase and phosphate‐buffered saline solution (PBS, pH 7.4, Thermo Fisher Scientific) was done using the NxGen microfluidic cartridge, under controlled flow conditions, at room temperature. The organic phase was an ethanolic solution of cholesterol (Chol), 1‐palmitoyl‐2‐oleoyl‐glycero‐3‐phosphocholine (POPC), and 1,2‐distearoyl‐sn‐glycero‐3‐phosphoethanolamine‐N‐[methoxy(polyethyleneglycol)‐2000] (DSPE‐PEG2000) at a molar ratio of 45:54.8:0.2, respectively, with a total lipid concentration of 10 mg/mL. A total flow rate of 12 mL/min was applied at an organic: aqueous phase ratio of 1.5:1. The first 0.35 mL and the last 0.05 mL of the resulting mixture were discarded, resulting in a batch of about 2 mL. This was dialyzed for 24 h in PBS at room temperature in a dialysis cassette (Pur‐A‐Lyzer dialysis kit, regenerated cellulose, 3.5 kDa) to remove ethanol. The resulting liposome suspension was then post‐inserted with DSPE‐PEG2000 to reach a final dose of 3 mol%. The storage of liposomes was at 4°C.

Compact, mRNA LNP particles were formulated with a lipid composition KC2/DSPC/cholesterol/PEG‐DMG (50/10/38.5/1.5 mol%) in 25 mm NaOAc with luciferase mRNA (N/p = 6) as described elsewhere [33].

Poly A‐loaded LNPs were also prepared using the Nanoassemblr Ignite microfluidic synthesizer instrument. The lipid mixture solution contained ALC0315, cholesterol, 1,2‐distearoyl‐sn‐glycero‐3‐phosphocholine (DSPC), and DSPE‐PEG‐2000 at a molar ratio of 50:38.5:10:1.5 in ethanol. All lipids were purchased from Avanti Research (Alabaster, AL, USA). PolyA (Sigma‐Aldrich; Burlington, MA, USA) was diluted in Acetate Buffer 20 mm at a final concentration of 80 µg/mL. Lipid mixture and RNA were mixed by applying the NxGen microfluidic cartridge at a flow rate of 12 mL/min and an aqueous‐to‐organic flow rate ratio of 3:1. The first 0.45 mL and the last 0.05 mL of the resulting mixture were discarded. Ethanol was removed from the synthesis buffer by washing the particle suspension with PBS applying centrifugation at 2000 xg (on a swing bucket rotor) in a 10 kDa molecular‐weight‐cut‐off Amicon filter (EMD Millipore, Billerica, MA, USA). The formulation was sterilized by filtering it through a 0.22 µm Supor Membrane Low‐Protein‐Binding Acrodisc Syringe Filter and stored at 4°C until use.

### 4.3 Dynamic Light Scattering (DLS)

DLS measurements were run using a Zetasizer Nano ZS (Malvern Instruments, Worcestershire, UK). Liposome suspensions were diluted five times, LNP suspensions were diluted 20 times with PBS, and analyzed after 180 s equilibration time in single‐use, PMMA semi‐micro cuvettes (BRAND UV cuvette micro, Merck), at 25°C.

### 4.4 Analytical Ultracentrifugation

Sedimentation velocity measurements were done in a Proteomelab XL‐I analytical ultracentrifuge equipped with both absorbance and interference optics (Beckman Coulter, Indianapolis, IN, US) using an 8‐hole titanium rotor, in 2‐sector centerpiece, sapphire window cells.

Sedimentation of polystyrene particles in various D_2_O‐containing solvents was observed at 30 000 rpm. Liposomes and lipid nanoparticles were run in the various liquid media at 10 000 rpm, in three repetitions. The final dilution of PS particles was 20x, while for liposomes and mRNA LNPs a final dilution of 40x was applied. The sedimentation of PS particles was monitored using the interference optics, and liposomes and lipid nanoparticles were analysed based on absorbance measurements run at 220 nm and 260 nm, respectively.

### 4.5 Calculations

#### 4.5.1 Model Fitting

Sedfit v17 was used for data analysis. The ls‐g*(s) model was applied for all particles, fitting or the meniscus or cell bottom position depending on the sedimentation behavior of the main particle population [36]. In case of PS particles, interference data were fitted in the −200 to 200 S range at a resolution of 200. For liposomes and lipid nanoparticles a wider range (−800 to 200 S) and absorbance data (at 220 and 260 nm) were used at a resolution of 200. Before the transformation of sedimentation coefficient distribution data to size distributions, Lamm equation calculations were re‐run in a positive (1–200 S) or negative (−1 to 200 S) range. Size distribution data generated in the negative sedimentation coefficient range by Sedfit were multiplied by −1 to generate positive size distributions. This step and all other theoretical calculations and data visualization were run in Excel (Microsoft Corporation, Redmond, WA, US).

#### 4.5.2 Particle Density

Calculations of particle density based on experiments in two different solvents were done in accordance with the ISO standard method [23] considering, Stokes law and applying the shape factor of one and the mode of the sedimentation coefficient distribution (s_1_ and s_2_) as sedimentation velocities in the equations.

(1) $$s_{1}=\frac{\left(\rho_{P}\;-\rho_{L,1}\;\right)\cdot D^{2}\;}{18\cdot \eta_{1}}\;$$

(2) $$s_{2}=\frac{\left(\rho_{P}\;-\rho_{L,2}\;\right)\cdot D^{2}\;}{18\cdot \eta_{2}}\;$$

where,

s_1_ is the sedimentation coefficient in liquid 1

s_2_ is the sedimentation coefficient in liquid 2

ρ_P_ is particle density

ρ_L_ is liquid density

D is particle diameter (equivalent sphere diameter)

η is liquid viscosity

(3) $$\rho_{P}=\frac{\;s_{1}\cdot \eta_{1}\cdot \rho_{L,2}-\;\cdot s_{2}\cdot \eta_{2}\cdot \rho_{L,1}\;}{s_{1}\cdot \eta_{1}-s_{2}\cdot \eta_{2}}\;$$

Liquid density and viscosity at 20°C for water and its mixtures with D_2_O were estimated by linear interpolation and are shown in Table S1.

Density and viscosity of the suspensions in 25% sucrose were calculated by linear interpolation considering the dilution factor and sucrose density and viscosity data at 20°C from literature.

#### 4.5.3 Theoretically Predicteds Distributions

Theoretical calculations to generate sedimentation coefficient distributions in D_2_O based on the distributions observed in aqueous buffer were done by generating cumulative *sedimentation coefficient distribution*s. Then *s*_2,n_ values that would correspond to the same particle sub‐population (n) in D_2_O were calculated from *s*_1,n_ (aqueous) values using the particle densities determined from the experiment in sucrose.

(4) $$s_{2,n}=\frac{s_{1,n}\;\left(\rho_{P}\;-\rho_{L,2}\;\right)\cdot \eta_{1}\;}{\eta_{2}\;\left(\rho_{P}\;-\rho_{L,1}\;\right)}\;$$

Then, differential distributions were generated from the cumulative ones using the s_2,n_ values.

#### 4.5.4 Particle Mass Increase

Partial specific volume of liposomes in the deuterated solvent was estimated using the Calculator function of Sedfitv17. The corresponding density was $\rho_{D_{2}O\mathrm{part}}$ = 1.074 g·cm^−3^. Thus, the observed mass increase of the particles was calculated by comparing this to the density of liposomes calculated from the ± sucrose/PBS experiment ($\rho_{H_{2}O\mathrm{part}}$ = 1.005 g·cm^−3^):

(5) $$(\rho_{D_{2}Opart/}\rho_{H_{2}Opart})*100-100=6.87\%$$

The expected mass increase when exchanging all H_2_O molecules in a 70 nm diameter (r = 35 nm radius) aqueous core to D_2_O was calculated as follows.

Diameter of the particles was considered to be 81.6 nm, which corresponds to a radius of R = 40.8 nm. Thus, the mass of a liposome in an aqueous buffer is:

(6) $$m_{H_{2}O\;\mathrm{part}\;}=4\cdot R^{3}\pi \cdot \rho_{H_{2}O\;\mathrm{part}}\;$$

Mass of the core filled with water is:

(7) $$m_{H_{2}O\;\mathrm{core}}=4\;r^{3}\pi \cdot 0.988\;g\cdot {\mathrm{cm}}^{-3}$$

Mass of the lipid bilayer shell is:

(8) $$m_{\mathrm{shell}}=m_{H_{2}O\;\mathrm{part}}-\;m_{H_{2}O\;\mathrm{core}}$$

Mass of the core filled with D_2_O is:

(9) $$m_{H_{2}\mathrm{Ocore}}=4\;r^{3}\pi \cdot 1.1054\;g\cdot cm^{-3}$$

Mass of the particles in D_2_O‐based solvent is:

(10) $$m_{D_{2}O\mathrm{part}}=m_{H_{2}\mathrm{Ocore}}+m_{\mathrm{shell}}$$

The expected mass increase is:

(11) $$(m_{D_{2}O\;\mathrm{part}}/m_{H_{2}O\;\mathrm{part}})*100-100=6.74\%$$

### 4.6 Cryo‐Transmission Electron Microscopy (Cryo‐TEM)

Cryo‐TEM images of the liposomes and LNPs with water compartments were visualized using a JEOL transmission electron microscope (JEM‐2100, JEOL, Italy) at 120 kV in cryo‐TEM mode. After pre‐concentration to about 15–20 mg·cm^−3^ total lipid concentration, sucrose solution was added to the sample suspensions at a final concentration of 10%, followed by the deposition on a glow discharge pretreated (10 mV, 30 s. Leica EM ACE 200, Microcontrol, Italy) formvar holey carbon film grid, and plunge freezing of the samples was performed (Leica EM GP, Italy). Grids were inserted in the microscope using a Gatan model 626 single tilt liquid nitrogen cryo transfer holder.

Cryo‐TEM image of the compact LNPs was collected using a FEI LaB6 G2 TEM (FEI, Hillsboro, OR) microscope equipped with an FEI Eagle 4 K CCD camera at 200 kV under low‐dose conditions. After concentrating the samples to about 20–25 mg·cm^−3^ total lipid concentration, a 3–5 µL aliquot was deposited on a glow‐discharged copper grid and plunge‐frozen using an FEI Mark IV Vitrobot (FEI, Hillsboro, OR, USA) to generate vitreous ice. Grids were inserted in the microscope using a Gatan 70° cryo‐tilt transfer system after pre‐equilibration to at least −180°C.

### 4.7 Statistical Analysis

Dynamic light scattering measurement results are presented by averaging distributions resulting from *n* = 3 measurements.

Nanoparticle tracking analysis results are presented as number‐weighted size distribution generated as an average of *n* = 5 measurements.

Fitting of analytical ultracentrifugation‐based mRNA LNP and liposome data considered the number of time points (measurements) that allowed for observing close to complete floatation or sedimentation of the particles (*n* = 25–100, depending on sample properties Figure S4). All data fits were done applying a confidence level value of 0.95.

## Funding

This study has received part of its funding from the European Union's Horizon 2020 research and innovation program under grant agreement No. 101007417 within the framework of the Nanoscience Foundries and Fine Analysis (NFFA)‐Europe activity, proposal ID725. This study was also supported by the European Commission Joint Research Centre – Exploratory Research Program Call 2023, project IDs: PANGOLIN and TARGET‐RNA, hosted by F.2 Unit ‐Technologies for Health in the JRC Nanobiotechnology Laboratories at JRC – Ispra M.H.Y.C was supported by CIHR Research Excellence, Diversity, and Independence (REDI) Early Career Transition Award (AWD‐031113). I.L. and S.C.D.S acknowledge support from European Union's Horizon 2020 Project BAXERNA 2.0 (101080544). Funded by the European Union (ERC, My‐NANO, 101230424) awarded to I.L. Views and opinions expressed are however those of the author(s) only and do not necessarily reflect those of the European Union or the European Research Council. Neither the European Union nor the granting authority can be held responsible for themHorizon Europe ERC.

## Conflicts of Interest

The authors declare no conflicts of interest.

## Supporting information




**Supporting File**: smtd70798‐sup‐0001‐SuppMat.docx.

## Data Availability

Raw data are available at https://data.jrc.ec.europa.eu/dataset/f48f6497‐c949‐4feb‐ac5f‐fd156b7389d0.
