# Supplementary material for: Analytical Ultracentrifugation in Different High‐Density Media Allows to Assess Heterogeneity of mRNA‐Lipid Nanoparticles
Source: Small Methods. 2026 Jun 26;10(14):e02420. doi: 10.1002/smtd.202502420 (PMC13397270; doi:10.1002/smtd.202502420)
Supplement: Supplementary file 1 — Supporting File: smtd70798‐sup‐0001‐SuppMat.docx. [file SMTD-10-e02420-s001.docx]

Supporting Information

Analytical ultracentrifugation in different high-density media allows to assess heterogeneity of mRNA-lipid nanoparticles

Dora Mehn*, Mariana Hugo Silva, Rein Verbeke, Allegra Peletta, Miffy Hok Yan Cheng, Stefaan C. De Smedt, Silvia Lucia Appleton, Ambra Sarracino, Jessica Ponti, Luigi Calzolai, and Ine Lentacker

**Table S1.** Sedimentation coefficient of polystyrene particles in various D_2_O containing solvents

| D_2_O [%]* | Density  ρ [g∙cm^-3^]* | viscosity  η [mPa∙s]* | s [S]** | s∙η [S∙mPa∙s]*** |
| --- | --- | --- | --- | --- |
| 0 | 0.998 | 1.002 | 111.6 | 111.8 |
| 25 | 1.025 | 1.064 | 53.3 | 56.7 |
| 50 | 1.052 | 1.127 | 5.03 | 5.67 |
| 60 | 1.062 | 1.152 | -13.1 | -15.0 |
| 70 | 1.073 | 1.177 | -31.2 | -36.7 |
| 80 | 1.084 | 1.202 | -47.2 | -56.8 |
| 95 | 1.100 | 1.239 | -73.4 | -90.9 |

* nominal values

** measured values (min n=54 time points),

*** calculated value


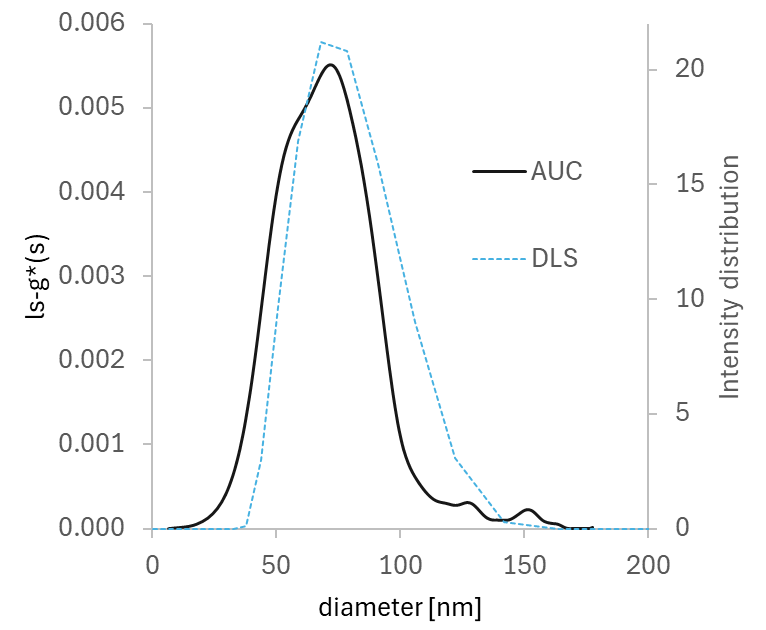


**Figure S1.** AUC and DLS-based size distribution of LNPs with aqueous cavities showed good agreement with the size distribution calculated from particle density measurements obtained in the +/- sucrose/PBS experiments. (Average of 3 measurements, n=3)

**Sedimentation of liposomes in H_2_-^18^O based solvent**

Sedimentation velocity measurements with a second batch of liposomes (synthesized as described in Section 4. Experimental methods) were done in a Beckman Coulter Optima analytical ultracentrifuge equipped with both absorbance and interference optics (Beckman Coulter, Indianapolis, IN, US) using an 8-hole titanium rotor, in 2 sector centerpiece, sapphire window cells.

H_2_-^18^O (99%) was purchased from Merck. PBS/ H_2_-^18^O was prepared by dissolving 50 mg of homogenized PBS tablet in 5 mL of the solvent. The liposome suspension was diluted 40x in this solvent. PBS/ H_2_-^18^O was loaded in the reference sector of the 2 sector sample cells. All other measurements and data evaluation were done as described in the Analytical Ultracentrifugation and Calculations subsections of Section 4. Experimental methods. All experiments were run in triplicates. Fits were done for all solvents in the -200 to 200 S range at 200 resolution and fitting the meniscus or the bottom position depending on the movement direction (floatation or sedimentation) of the particles. The mode of the distribution was considered as the sedimentation coefficient of the main population (average of three measurements, n=3).


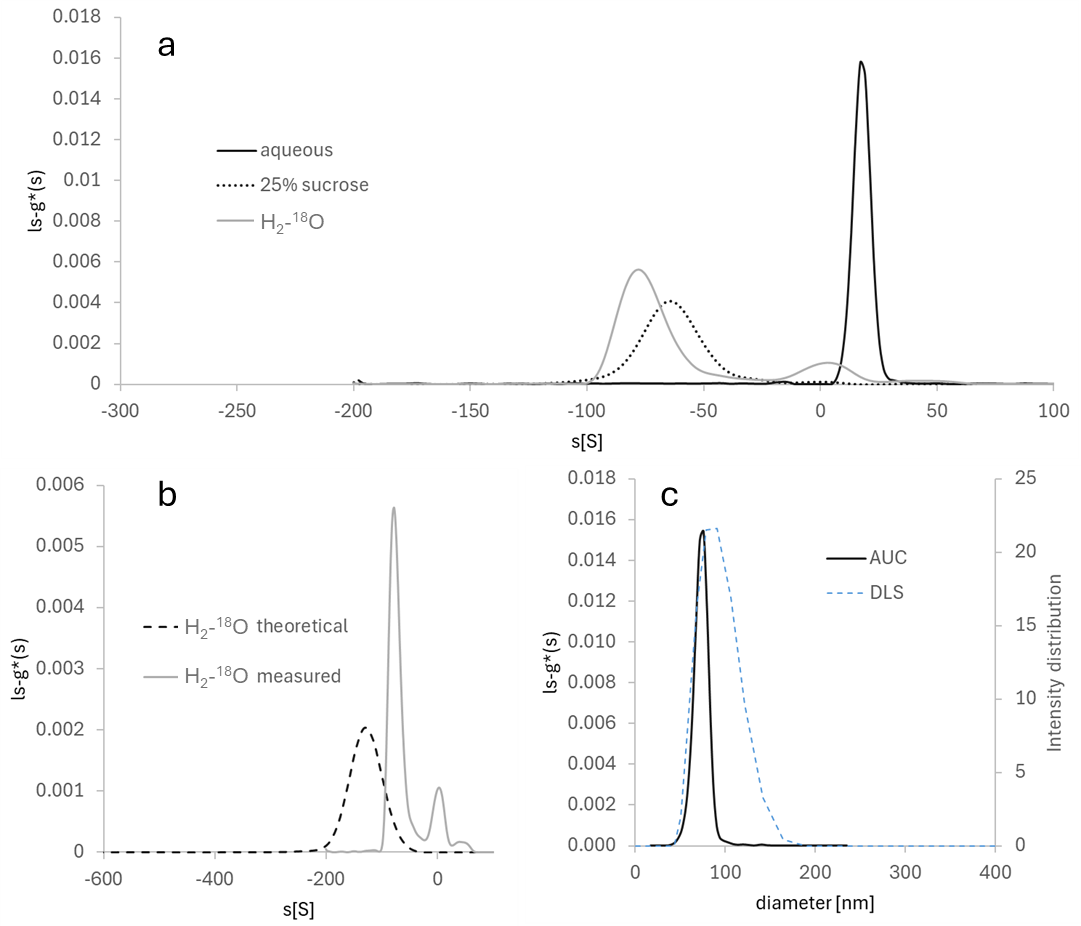


**Figure S2**. a) Sedimentation coefficient distribution of liposomes in aqueous buffer (solid, black line), 25% sucrose solution (dotted line) and H_2_-^18^O-based buffer (grey line); b) Sedimentation coefficient distribution of liposomes measured in H_2_-^18^O-based buffer (grey line) compared to the theoretically expected distribution calculated from the sedimentation profile in aqueous buffer (dashed line). Note, that the measurement in H_2_-^18^O is the same as in Figure 2a, only scales are different; c) Hydrodynamic size distribution calculated from AUC results in aqueous buffer and in sucrose containing medium (solid, black line) compared to the size distribution measured with DLS (dotted blue line). (n=3, confidence level 0.95)

As illustrated in Figure S2 the mode of the sedimentation coefficient distribution of these liposomes appeared at 17S and was shifted to about -64S in 25% sucrose. In H_2_-^18^O, the mode appeared at about -77S – suggesting that the particles floated in this solvent slower than the theoretically expected velocity (about -130S). The difference – also in this case – can be attributed to the solvent exchange in the aqueous cavity of the particles. Model calculations applying the density of H_2_-^18^O containing solvent for the particle core resulted in an expected coefficient of -79S – in quite good agreement with the observed -77S value.

The small, very slowly sedimenting additional particle population appearing at about 7S in H_2_-^18^O has a calculated diameter of about 15 nm - if we suppose that these particles do not have a cavity that would change density in the higher density medium and that their signal is contributing to the peak observed at 17S in aqueous buffer. This size corresponds to the expected size of DSPE-PEG2000 micelles, that were used for the post-synthetic modification of the liposome batch (as described in Section 4. Experimental methods) and might be residual micelles still present after purification. Figure S3 illustrates their size distribution measured by DLS at 14.17 mg/mL concentration in PBS.


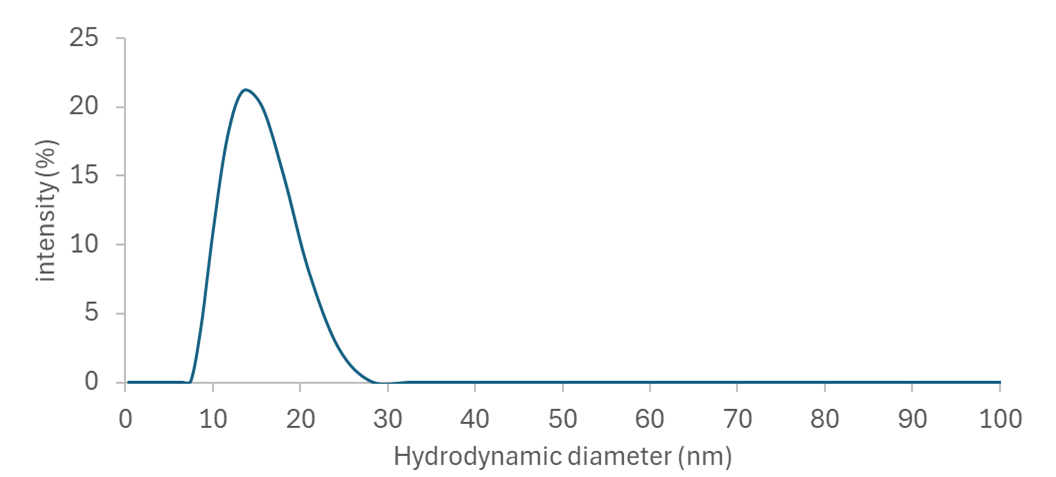


**Figure S3**. Hydrodynamic diameter of the DSPE-PEG2000 micelles used for post-synthetic modification of the liposome sample (n=3).

**Comparison between sizing methods**

The same batch of liposomes was used to generate an example comparison between various sizing methods. Dynamic light scattering (DLS) measurements were performed as described in the Section 4. Experimental methods of the article. Nanoparticle tracking analysis (NTA) of these particles was performed using a Nanosight NS500Z instrument equipped with a 405 nm light source. The particle suspension was diluted 30000x in 0.2 µm filtered PBS and injected in the measurement flow cell by using the peristaltic pump of the instrument. After temperature equilibration at 25 °C and setting the focus, three 60s long records were acquired (Camera level setting of 11), approximately five different aliquots of the sample, by automatically advancing the suspension in the flow cell between measurements. The collected records were processed using the NTA 3.4 Build 3.4.003 software version by applying detection threshold value of 2 and automatic analysis settings.

As illustrated in Figure S4, different sizing methods provide slightly different size distribution results for the same PEGylated liposome sample. DLS used with default settings suggested an intensity-based distribution with a mode shifted to higher sizes compared to our AUC method (that considers sedimentation speed distributions in aqueous buffer and in the presence of 25% sucrose).


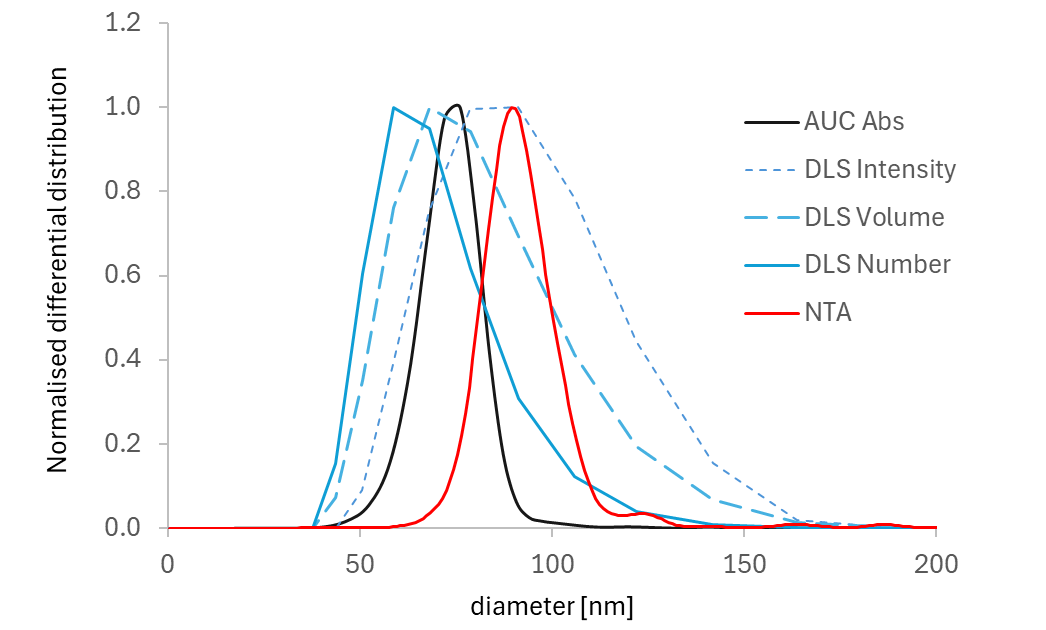


**Figure S4.** Size distribution of the same liposome batch measured by various sizing methods (AUC and DLS n=3, NTA n=5)

Volume-based distribution (considering a refractive index of 1.45 and absorption of 0.001) shows a better match with AUC data, while the number-based distribution is shifted to lower values. Surprisingly, NTA provided a size distribution with a higher mode than AUC (and the number weighted results from DLS). After careful verification of the instruments’ performance with polystyrene beads, we attribute this unusual result to the weak light scattering properties of these liposomes. NTA provides number weighted results, but it is still affected by the light scattering properties of the particles that show exponential size dependence in this size range. While metallic particles (such as gold) can be measured with NTA down to about 30 nm diameter, in case of lipid-based systems - like liposomes and LNPs - the limits of the capabilities are reached at about 80 nm. The missed detection of smaller particles results in a bias in the final size distribution.

**Examples of fit screenshots**

Analytical ultracentrifugation data fits were all done using Sedfit as described in the Section 4. Experimental methods of the article. The s_min_ and s_max_ limits were adjusted to cover the specific range of the individual sample types, resolution was kept at 200 and the confidence level (F ratio) was set to 0.95. Examples are shown in Figure S5.


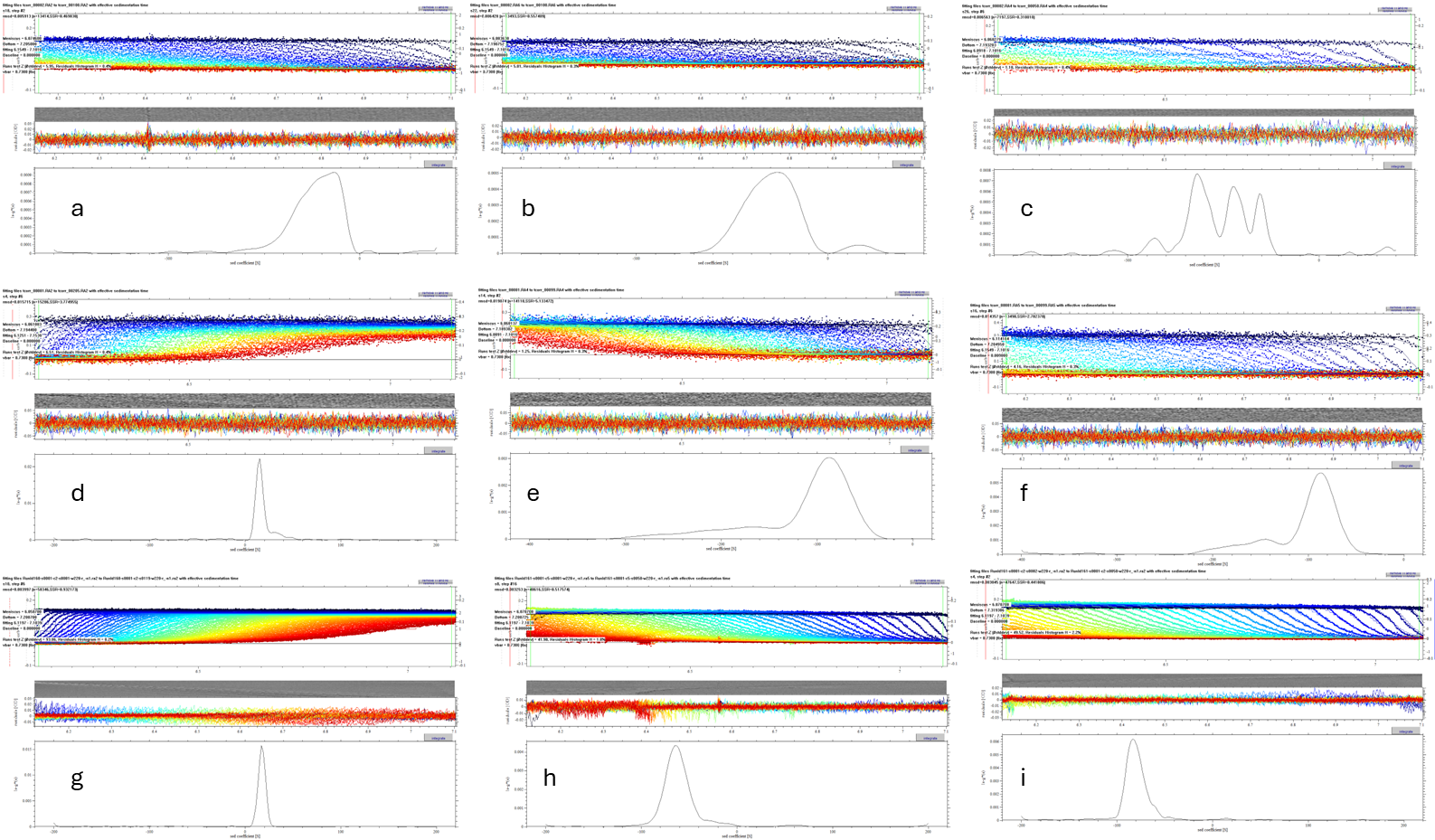


**Figure S5.** Screenshot examples of fits for mRNA LNPs (a,b,c) and two batches of PEGylated liposomes (d,e,f and g,h,i). First column: aqueous PBS buffer; Second column: 25% sucrose/PBS; Third column: heavy waterbased solvents c,f deuterated water, i.e. H_2_-^18^O.
